# Development of a gene cloning system in a fast-growing and moderately thermophilic *Streptomyces *species and heterologous expression of *Streptomyces *antibiotic biosynthetic gene clusters

**DOI:** 10.1186/1471-2180-11-243

**Published:** 2011-10-28

**Authors:** Weihua Chen, Zhongjun Qin

**Affiliations:** 1 Key Laboratory of Synthetic Biology, Shanghai Institute of Plant Physiology and Ecology, Shanghai Institutes for Biological Sciences, the Chinese Academy of Sciences, 300 Fenglin Road, Shanghai, 20032, People's Republic of China

## Abstract

**Background:**

*Streptomyces *species are a major source of antibiotics. They usually grow slowly at their optimal temperature and fermentation of industrial strains in a large scale often takes a long time, consuming more energy and materials than some other bacterial industrial strains (e.g., *E. coli *and *Bacillus*). Most thermophilic *Streptomyces *species grow fast, but no gene cloning systems have been developed in such strains.

**Results:**

We report here the isolation of 41 fast-growing (about twice the rate of *S. coelicolor*), moderately thermophilic (growing at both 30°C and 50°C) *Streptomyces *strains, detection of one linear and three circular plasmids in them, and sequencing of a 6996-bp plasmid, pTSC1, from one of them. pTSC1-derived pCWH1 could replicate in both thermophilic and mesophilic *Streptomyces *strains. On the other hand, several *Streptomyces *replicons function in thermophilic *Streptomyces *species. By examining ten well-sporulating strains, we found two promising cloning hosts, 2C and 4F. A gene cloning system was established by using the two strains. The actinorhodin and anthramycin biosynthetic gene clusters from mesophilic *S. coelicolor *A3(2) and thermophilic *S. refuineus *were heterologously expressed in one of the hosts.

**Conclusions:**

We have developed a gene cloning and expression system in a fast-growing and moderately thermophilic *Streptomyces *species. Although just a few plasmids and one antibiotic biosynthetic gene cluster from mesophilic *Streptomyces *were successfully expressed in thermophilic *Streptomyces *species, we expect that by utilizing thermophilic *Streptomyces*-specific promoters, more genes and especially antibiotic genes clusters of mesophilic *Streptomyces *should be heterologously expressed.

## Background

*Streptomyces *species are high G+C, Gram-positive bacteria that are a major source of natural products, producing about half of all known microbial antibiotics [1]. Members of this genus also have a complex life cycle, in which uni-genomic spores geminate to produce a multi-genomic substrate mycelium of branching hyphae which gives rise to aerial hyphae and ultimately to spores [2]. *Streptomyces coelicolor *A3(2) is the genetically most studied *Streptomyces *species from the *in vivo *through *in vitro *to *in silico *phases and is an excellent model for studying antibiotic production and differentiation [3,4]. Mainly because of a strong restriction barrier to introduction of foreign double-stranded DNA by transformation from *Escherichia coli *into A3(2), the closely related *S. lividans*, with no such barrier and cured of indigenous plasmids (SLP2 and SLP3: [5]), has been used as a standard host for gene cloning and expression for several decades [6]. However, compared with *E. coli *and *Bacillus subtilis*, *S. coelicolor *and *S. lividans *(also other species from the genus *Streptomyces*) grow slowly at their optimal temperature (e.g., *S. coelicolor *M145 - a plasmid-free derivative of A3(2) - grows exponentially with a doubling time of about 2.2 h on SMM medium at 28°C, see ref [6]). It takes about 2-3 weeks for *Streptomyces *strains to produce and accumulate antibiotics at a good yield on an industrial scale.

Fast-growing, thermophilic *Streptomyces *strains have been studied for a long time. Some earlier described thermophilic *Streptomyces *species (e.g., *S. thermophilis *and *S. thermofuscus*: [7,8]) were not classified as thermophilic streptomycetes [9,10]. Numerical classification of thermophilic streptomycetes showed three major, five minor and two single-member clusters [10]. Analysis of the 16S rRNA genes and morphological and chemical properties indicate their classification within the genus *Streptomyces *[11,12]. Most thermophilic *Streptomyces *species have growth temperature ranges from 28 to 55°C and so are only moderately thermophilic [11,12]. However, some thermophilic *Streptomyces *species can grow up to 68°C [13]; the optimum growth temperature of *S. thermoautotrophicus *is 65°C and no growth is observed below 40°C, so it is a truly thermophilic strain [14]. Growth of thermophilic *Streptomyces *strains is rapid at high temperature [15]; for example, *S. thermoviolaceus *has a doubling time of 1 h at 50°C [16]. Thermophilic *Streptomyces *species produce thermostable enzymes and antibiotics [15], such as xylanase [17], alpha-amylase [18], granaticin [16] and anthramycin [19]. Since thermophilic *Streptomyces *strains lack a genetic manipulation system, mesophilic strains (e.g. *S. lividans*) have been employed for expression of some genes or antibiotic biosynthetic gene clusters from thermophilic *Streptomyces *species [20-22].

We report here the development of a gene cloning system in a fast-growing (about twice the rate of *S. coelicolor*) and moderately thermophilic (growing at both 30°C and 50°C) *Streptomyces *strain, and successful heterologous expression of antibiotic biosynthetic gene clusters from both thermophilic and mesophilic *Streptomyces *species.

## Results and Discussion

### Isolation and identification of thermophilic *Streptomyces *strains from various soil samples

To isolate thermophilic *Streptomyces *strains, various soil samples from China were collected (see Methods). As summarized in Table 1, 22, 11 and eight strains were isolated from samples of garden soil, weed compost and swine manure, respectively. Thermophilic *Streptomyces *species have been isolated from composts, soil and sewage [23], as well as lakes and hot-springs [13]. Our results reinforce the idea of a widespread occurrence of these organisms.

**Table 1:** Strains used in this study

Strains	Genotype or description	Source or reference
*Streptomyces *		
*S. coelicolor *M145	SCP1^- ^SCP2^-^	[6]
*S. lividans *1326	SLP2 SLP3	[6]
*S. lividans *ZX7	*pro-2 str-6 rec-46 dnd *SLP2^- ^SLP3^-^	[37]
*S. venezuelae *ISP5230	A jadomycin B producer	[49]
Thermophilic *Streptomyces*		
22 strains (4F, 2C, T1-1, T1-2, T5A-1, T6-5, T6-1-4, T1A, T6A-2, T6A-3, T6B-1, T6B-2, T6B-5, T6B-6, T6B-8, T6C-1, T6C-5, T6D-1, T6D-2, T6E-1, T6E-2, T6F-2)	Isolated from garden soil at the Shanghai Institute of Plant Physiology and Ecology	This work
11 strains (G101, G103, G303, G102, G507, G302, 403, 202G, G304, 301, 005)	Isolated from weed manure in Hunan province	This work
8 strains (A1, Z2-1, X1-3, X1-6, X2-1, X3-3, X4-3, X5-5)	Isolated from swine manure in Hunan, Hubei and Fujian province	This work
*Escherichia coli*		
DH5α	*F- deoR recA1 endA1 hsdR17(rk- mk+) phoA supE44 λ- thi-1 gyrA96 relA1*	Life Technologies, Inc
ET12567 (pUZ8002)	*dam dcm hsdM cm kan*	[6]

**Figure 1:**
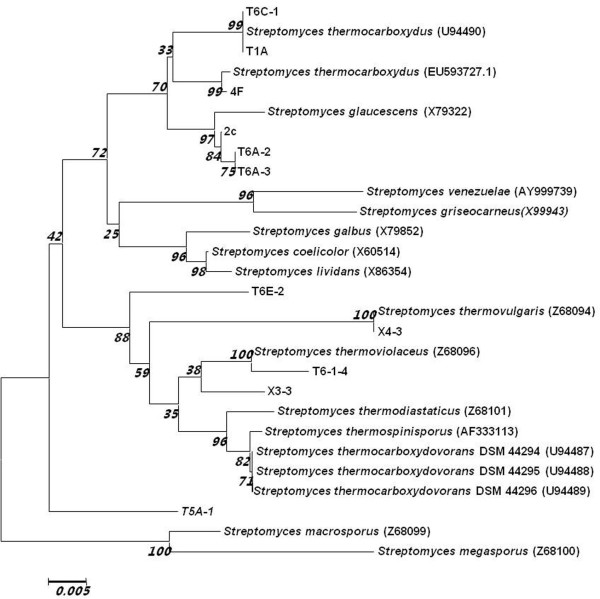
**Identification of thermophilic *Streptomyces *strains**. Phylogenetic tree for 11 newly identified strains and some known mesophilic and thermophilic *Streptomyces *species (Genbank numbers in parentheses). The tree is drawn to scale using the neighbor-joining method, with branch lengths in the same units as those of the evolutionary distances. Numbers next to the branches are the percentage of replicate trees (the bootstrap test is 500 replicates).

To determine if these strains should be classified in the genus *Streptomyces*, 16S rRNA genes were amplified, cloned and sequenced. The sequences displayed high homology (97-99%) to those of known mesophilic *Streptomyces *and/or thermophilic *Streptomyces *species. A phylogenetic tree was drawn by using a neighbor-joining method [24]. The chosen 11 newly isolated strains have distinct phenotypes when cultured on R2YE and MS media, and the reference strains utilized for comparison are well-classified *Streptomyces *species. As shown in Figure 1, all 11 newly isolated strains (4F, T6C-1, T1A, T6E-2. X4-3, T6-1-4, X3-3, 2C, T5A-1, T6A-2 and T6A-3) resembled known thermophilic *Streptomyces *species (e.g., *S. thermocarboxydus*, *S. thermoviolaceus *and *S. glaucescens*). Moderately thermophilic *Streptomyces *species form at least two distinct clades [12,23,25], containing strains related to *S. megasporus *and *S. thermodiastaticus*, respectively. The phylogenetic tree of the 11 newly isolated strains reveals more clades (e.g., T5A-1 and T6E-2; see Figure 1). These results indicate that moderately thermophilic *Streptomyces *species are diverse in natural habitats.

Like typical *Streptomyces *species, these newly isolated strains produced spores on R2YE and MS media. Scanning electron microscopy showed that strains 4F and 2C formed long chains of smooth-surfaced spores after growth on MS medium at 42°C for 2 d (data not shown). Thus strains 4F and 2C were classified in the genus *Streptomyces*.

### Characterization of the fast-growing and moderately-thermophilic *Streptomyces *strains 4F and 2C

**Figure 2:**
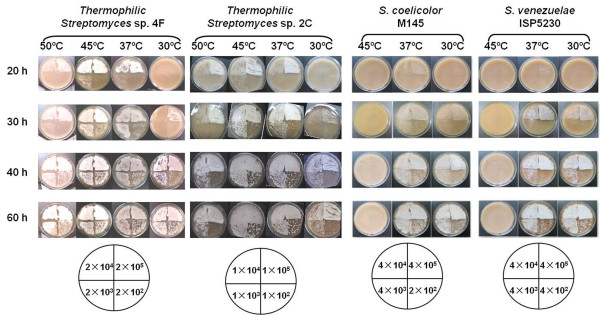
**Growth of strains 4F, 2C, M145 and ISP5230 on MS medium at different temperatures in a time-course**. A series of 10× dilutions of spore suspensions were inoculated onto MS medium and incubated at 30, 37, 45 and 50°C in a time-course at 20, 30, 40 and 60 h. The numbers of spores of the four strains inoculated on plates are shown. 4F and 2C were cultured at 30, 37, 45 and 50°C, while M145 and ISP5230 were grown at 30 37 and 45°C.

As shown in Figure 2, strains 4F and 2C were able to grow from 30 to 50°C, while two mesophilic *Streptomyces *strains (*S. coelicolor *M145 and *S. venezuelae *ISP5230) grew at 30°C and 37°C. 4F and 2C grew well at 45°C and 50°C but poorly at 55°C, while M145 and ISP5230 could not grow at 45°C and 50°C (data not shown). Thus, 4F and 2C were concluded to be moderately thermophilic *Streptomyces *strains.

**Figure 3:**
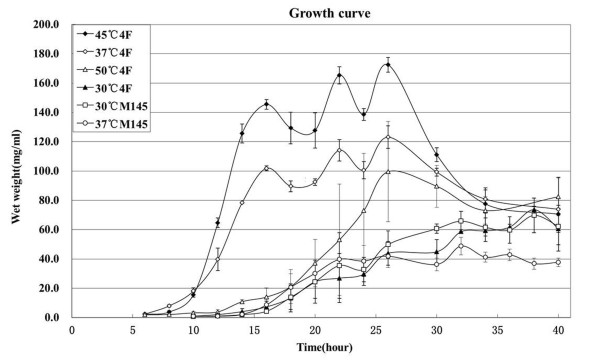
**Growth curves of 4F and M145 in liquid culture at four temperatures**. The curves are based on the average of three weighings at each time point, and standard deviations are indicated.

Strains 4F and 2C grew on MS medium at 37°C and 45°C faster than the mesophilic *Streptomyces *strains at 30°C and 37°C (Figure 2). To measure the growth rates of 4F and M145, equal numbers of spores were inoculated into TSB liquid medium, and three mycelial samples were harvested at various points during the time course. Each sample was weighed, and the three values were averaged for a particular time point. As shown in Figure 3, 4F rapidly accumulated biomass to a maximum at 45°C or 37°C within 16 h, then the growth curve fluctuated, and the final biomass of strain 4F is higher for M145 (especially at 45°C). The oscillations shown at 37 and 45°C resembling the "death/growth process" of *S. coelicolor *A3(2) in liquid medium with a diluted inoculum [26]. The doubling times of growth for 4F at 30, 37, 45 and 50°C and M145 at 30°C and 37°C in each logarithmic phase (14-20, 6-12, 8-14 and 12-18 h for 4F at 30, 37, 45 and 50°C, and 16-22 for M145 at 30 and 37°C) were 2.3, 1.4, 1.1 2.3, 2.2 and 2.4 h, respectively. Thus strain 4F grew at 45°C twice and at 37°C 1.6 times as fast as M145 at 30°C in TSB medium.

**Figure 4:**
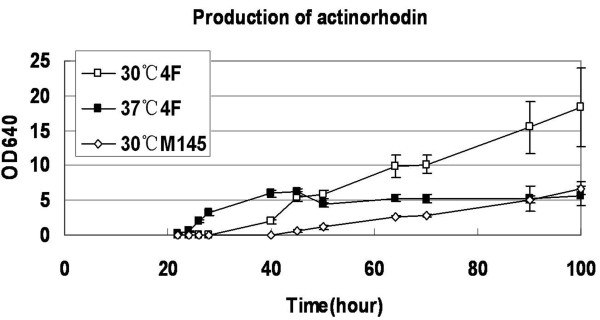
**Quantitation of actinorhodin production by M145 and by 4F containing the cloned actinorhodin gene cluster in liquid medium**. About 1 × 10^6 ^spores of M145 and of 4F containing pCWH74 were inoculated into 50 ml R2YE liquid medium (lacking KH_2_PO_4 _and CaCl_2_) at 30 and 37°C. Samples of 1 ml culture were harvested in a time-course and treated with KOH; absorption at OD640 indicated actinorhodin production.

### Identification of one linear and three circular plasmids among 41 strains, and sequencing of pTSC1

We detected three circular plasmids, 7-kb pTSC1, from X4-3, 7.5-kb pTSC2 from X3-3, and 40-kb pTSC3 as well as 16-kb linear pTSL1 from T6-1-4. The complete nucleotide sequence of the circular pTSC1 consisted of 6996 bp (GenBank accession number GU271942), with 72% G+C, resembling that of a typical *Streptomyces *genome (e.g., 72.1% for *S. coelicolor *A3(2): [27]). Eight ORFs (open reading frame) were predicted by "FramePlot 3.0 beta" [28]; seven of them resembled *Streptomyces *or *Mycobacterium *genes (Additional file 1, Table S1). Notably, three genes resembled the transfer and spread genes (*tra *and *spd*) of *Streptomyces *plasmids pIJ101 [29] and pSNA1 [30].

### Development of a gene cloning system in strains 2C and 4F

Followed the standard protocols of preparation and transformation of *Streptomyces *protoplasts with slight modifications (see Methods), pTSC1-derived pCWH1 (see Methods and Table 2) was introduced by transformation into ten well-sporulating thermophilic *Streptomyces *strains. Thiostrepton-resistant colonies were obtained for strains 2C and 4F at frequencies of 1.3 × 10^3 ^and 2 × 10^1 ^per μg DNA, but no transformants arose for the other eight strains. Many *Streptomyces *selection markers (e.g., *tsr*, *apr, spec*, *hyg*, *erm *and *kan*) could be used in strains 2C and 4F. No antibacterial activity (e.g., against *Bacillus subtilis*, *Escherichia coli *or *Staphyloccocus aureus*) was detected in the two strains (unpublished data). Thus, we found two promising cloning hosts, 2C and 4F.

**Table 2:** Plasmids used in this study

Plasmids	Genotype or description	Source or reference
pTSC1	A 6996-bp plasmid of strain X4-3	This work
pTSC2	A 7.5-kb plasmid of strain X3-3	This work
pTSC3	A 50-kb plasmid of strain T6-1-4	This work
pTSL1	A 16-kb linear plasmid of strain T6-1-4	This work
pSP72	*amp colEI-ori*	Life Technologies, Inc
pBluescript II SK	*amp colEI-ori lacZ*	Stratagene, Inc
pQC156	A 2.6-kb *Bcl*I-fragment of *melC/tsr *cloned in pSP72 (*Bgl*II)	[46]
pCWH1	A 7-kb *Kpn*I fragment of pTSC1 cloned in pQC156	This work
pCWH100	A 7-kb *Kpn*I fragment of pTSC1 cloned in pBluescript II SK	This work
pIJ702	*melC tsr *pIJ101 origin	[31]
pZR10	A 8.9-kb *Sau*3A1-fragment of pFP11 origin cloned in pQC156	[33]
pZR115	A 4.1-kb *Sau*3A1-fragment of pFP1 origin cloned in pQC156	[33]
pZR205	Two fragments (PCR) of SLP1 *rep/imp *cloned in pQC156	[33]
pZR51	A 2.2-kb *Hin*dIII fragment of pFRL2 origin cloned into pQC156	[32]
pHAQ61	A 2.9-kb fragment of SAP1 origin cloned in pQC156	Zhang and Qin, unpublished data
pYQ40	A 2-kb fragment of SCP2 origin cloned in pQC156	Yang and Qin, unpublished data
pGP9	A 4.1-kb *Eco*RI/*Bgl*II fragment of pSHK1 origin cloned in pQC156	[32]
pSET152	*Streptomyces *phage φC31-derived integration vector, *apr^r^*	[38]
pHAQ31	*amp colEI-ori cos melC tsr *	[47]
Cosmid N7-85	pHAQ31 (*Bam*HI) containing c. 33 kb sequence (5510413-5543521 bp) from *S. coelicolor *A3(2)	This work
pCWH74	A 2.6-kb *Xba*I/*Nhe*I fragment containing the phiC31 integrase gene cloned in a pHAQ31-derived cosmid containing the actinorhodin biosynthetic gene cluster	This work
024CAO-3	The anthramycin biosynthetic gene cluster cloned into a cosmid CAO2	[22]

Since 2C and 4F were classified in the genus *Streptomyces*, several mesophilic *Streptomyces *vectors were employed for transformation experiments. As shown in Table 3, pIJ702 (a pIJ101 derivative, [31]), pZR51 (pFRL2, [32]), pZR115 (pFP1, [33]) and pZR10 (pFP11, [33]) were able to transform both 2C and 4F. No transformants were obtained for SCP2 [34], SLP1[35], SAP1 [36] and pSHK1 [32] derivatives (pYQ40, pZR205, pHAQ61, and pGP9, respectively). pCWH1 could also transform *S. lividans *ZX7 [37] at high frequency (10^4^/μg DNA). A *Streptomyces *integrating plasmid, pSET152 [38], could be introduced by conjugation from *E. coli *into many thermophilic *Streptomyces *strains (14 of 22 strains). Thus, pTSC1-derived pCWH1 can replicate in both thermophilic and mesophilic *Streptomyces *strains. On the other hand, several *Streptomyces *replicons, including circular plasmids pIJ101, pFP1 and pFP11 and linear plasmid pFRL2, were able to propagate in the thermophilic *Streptomyces *strains 2C and 4F, but no transformants were obtained for circular plasmids SCP2 and SLP1 and linear plasmids SAP1 and pSHK1.

**Table 3:** Transformation by plasmids of the moderately thermophilic *Streptomyces *2C and 4F

Plasmids	Replicons	Hosts	Transformation frequency (transformants/μg DNA)	
			
			2C	4F
pIJ702	pIJ101	*S. lividans *ZX7	1.3 × 10^6^	3 × 10^2^
pIJ702	pIJ101	2C	2.9 × 10^6^	8 × 10^1^
pIJ702	pIJ101	4F	1.4 × 10^5^	1.2 × 10^5^
pCWH1	pTSC1	*E. coli *DH5α	1.3 × 10^3^	2 × 10^1^
pZR51	pFRL2	*E. coli *DH5α	8.2 × 10^3^	1 × 10^1^
pZR115	pFP1	*E. coli *DH5α	1 × 10^2^	2 × 10^1^
pZR10	pFP11	*E. coli *DH5α	2 × 10^2^	1

Comparing the transformation frequencies of pIJ702 from different hosts in 2C and 4F, as shown in Table 3, similar high frequencies of transformation (2.9 × 10^6 ^and 1.3 × 10^6^) were obtained in 2C with pIJ702 from both 2C itself and the largely restriction-free *S. lividans *ZX7. Low frequencies of transformation (8 × 10^1 ^and 3 × 10^2^) were obtained in 4F with pIJ702 from 2C and ZX7, although a high frequency (1.2 × 10^5^) was obtained with plasmid DNA from the strain itself. These results indicated that strain 2C showed essentially no restriction barrier to the introduction of foreign double-stranded DNA from other *Streptomyces *species, whereas strain 4F had a strong restriction barrier. The evaluation of restriction barriers needs much more experimental data to be supported.

### Heterologous expression of the actinorhodin biosynthetic gene cluster of *S. coelicolor *A3(2) in strain 4F

Since several mesophilic *Streptomyces *plasmids functioned in thermophilic *Streptomyces*, we chose a phage phiC31-derived integrating plasmid pSET152 [38] which is inherited stably in other hosts to perform experiment on heterologous expression of antibiotic biosynthetic genes in thermophilic *Streptomyces *strains. By using PCR with eight primers from the actinorhodin biosynthetic genes (*sco5085-5092*), we found that no bands for strains 4F and 2C were detected on agarose gel after electrophoresis of the PCR products, indicating no such genes in the strains. We cloned the complete actinorhodin biosynthetic gene cluster from *S. coelicolor *A3(2) in an integrating plasmid (see Methods), and the resulting plasmid, pCWH74, was introduced by conjugation into eight newly isolated strains, including 4F and 2C. PCR amplification experiments with eight paired primers from SCO5085 to SCO5092 confirmed the presence of the actinorhodin genes in the clones of 4F and 2C. Blue pigment was observed for strain 4F on both R2YE and MS media at 30 and 37°C after growth for 1 d, but no blue pigment was seen at 45°C. 2C with the actinorhodin gene cluster did not produce visible blue pigment on R2YE or MS media. To confirm that the blue pigment was actinorhodin, 4F containing pCWH74 was cultured in R2YE liquid medium lacking KH_2_PO_4 _and CaCl_2 _and the supernatant was treated with KOH and scanned at 640 nm [39]. The same pattern of absorption peaks was detected for 4F as for *S. coelicolor *A3(2) (data not shown). Thus the actinorhodin biosynthetic gene cluster from the mesophilic *S. coelicolor *A3(2) was heterologously expressed in strain 4F at low temperature (30 and 37°C), but not at high temperature (45°C).

To quantitate the productivity of actinorhodin, equal amounts of spores of M145 and 4F containing pCWH74 were inoculated into R2YE liquid medium lacking KH_2_PO_4 _and CaCl_2_, and 1 ml culture was harvested in a time-course. As shown in Figure 4, actinorhodin was produced in 4F at both 30 and 37°C, earlier than in M145 at 30°C. At 100 h, productivity of actinorhodin in 4F at 30°C was ~2.8 times higher than in M145 at 30°C. Strains M145 and 4F grew better in TSB than in R2YE liquid media (data no shown), but no actinorhodin was detected when cultured in TSB medium at 30 and 37°C. Growth curves of the two strains in R2 lacking KH_2_PO_4 _and CaCl_2 _at 30°C showed that their biomass values were similar from 20 to 120 hours (data not shown). Thus, better growth of M145 and 4F in TSB medium (Figure 3) did not correlate with delayed and less production of actinorhodin in R2YE medium (Figure 4).

Like in 4F, M145 produced more actinorhodin in R2YE medium at 30°C than at 37°C, suggesting that expression of the actinorhodin biosynthetic genes might be temperature-dependent. Temperature-dependent antibiotic gene clusters have been reported in *Streptomyces*, for example, much higher productivity of validamycin A produced by *Streptomyces hygroscopicus *was found at 37°C than at 30°C [40]. We infer that by replacement of thermophilic-specific promoters, many single genes and especially antibiotic genes clusters of mesophilic *Streptomyces *should be heterologously expressed in the fast-growing and thermophilic *Streptomyces*.

### Heterologous expression of the anthramycin biosynthetic gene cluster of the thermophilic *S. refuineus *subsp. *thermotolerans *in strain 4F

**Figure 5:**
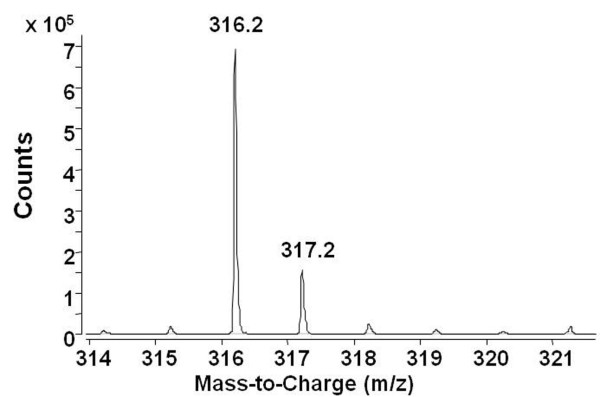
**Analysis of anthramycin production by HPLC/MS**. After separating anthramycin on an HPLC column, mass spectrometry was performed using 6520 Agilent Accurate-Mass Q-TOF LC/MS.

Expression of the anthramycin biosynthetic genes of *S. refuineus *subsp. *thermotolerans *could be detected at high temperature (i.e. 47°C), but not at 30 or 37°C [22]. An integrating cosmid, 024COA-3, containing the whole anthramycin biosynthetic gene cluster was introduced by conjugation from *E. coli *into strain 4F. PCR amplification experiments confirmed the presence of the anthramycin genes in the clone of 4F. After culturing in AP1 medium at 30, 37 and 47°C for 24 h, mycelium was extracted, dried and re-dissolved in MeOH. Thin-layer chromatography, followed by a bio-assay by overlaying with LB agar containing as indicator strain a *Bacillus *sp., revealed a zone of growth inhibition on 4F at 47°C, but no inhibition zone was found at 30 and 37°C (data not shown). A spot on a TLC plate was further purified for HPLC-MS analysis. As shown in Figure 5, an anthramycin-specific peak (ES+ = 316 Dalton, see ref [41]) was detected. Thus the anthramycin biosynthetic gene cluster of the thermophilic *S. refuineus *subsp. *thermotolerans *was heterologously expressed in strain 4F. We introduced the same cosmid 024COA-3 containing the anthramycin gene cluster into strain 2C, but no transformants were obtained. To see if strain 2C might be a better host than 4F, more antibiotic biosynthetic gene clusters should be tested.

## Conclusions

This study shows that by isolation of new strains and testing several plasmids, a host-vector system in a fast-growing and moderately thermophilic *Streptomyces *species could be developed. Two antibiotic biosynthetic gene clusters from mesophilic and thermophilic *Streptomyces *were heterlogously expressed in one strain. We expect that by utilizing thermophilic *Streptomyces*-specific promoters, more genes and especially antibiotic genes clusters of mesophilic *Streptomyces *should be heterologously expressed.

## Methods

### Bacterial strains, plasmids, and general methods

Strains used in this work are listed in Table 1. Plasmid isolation, transformation of *E. coli *DH5α and PCR amplification followed Sambrook *et al. *[42]. *Streptomyces *culture, plasmid isolation and preparation of protoplasts and transformation of *Streptomyces lividans *ZX7 followed Kieser *et al. *[6]. Plasmid trans-conjugation from *E. coli *ET12567/pUZ8002 into thermophilic *Streptomyces *strains followed Bierman *et al. *[38]. *Kpn*I-treated pTSC1 was cloned in pBluescript II SK to obtain pCWH100 and was sequenced by primer-walking at Shanghai Invitrogen Inc. Sequence comparisons were done with software from the National Center for Biotechnology Information http://www.ncbi.nlm.nih.gov/BLAST. The complete nucleotide sequence of pTSC1 was deposited in the GenBank database under no. GU271942.

### Isolation and identification of thermophilic *Streptomyces *strains

Samples of garden soil, weed compost and swine manure were collected from Shanghai city, Hunan, Hubei and Fujian provinces in the summers of 2005 and 2006. The samples were dried at 100°C for 1 h and cultivated on SC medium (starch 10 g, casein 0.3 g, KNO_3 _2 g, MgSO_4_.7H_2_O 0.05 g, FeSO_4_.7H_2_O 0.01 g, CaCO_3 _0.02 g, agar 18 g, H_2_O to 1000 ml, pH7.2) [43] at 50°C for 3-5 d. Thermophilic *Streptomyces *strains were cultured in TSB (Oxoid tryptone soya broth powder, 30 g, H_2_O to 1000 ml) liquid medium at 45°C for 1 d and genomic DNA was isolated followed the Kirby mix procedure [6]. 16S rRNA genes were amplified by PCR with primers (5'-AGAGTTTGATCCTGGCTCAG-3' and 5'-TCAGGCTACCTTGTTACGACTT-3'). PCR conditions were: template DNA denatured at 95°C for 5 min, then 95°C 30 s, 55°C 30 s, 72°C 2 min, for 35 cycles. PCR products were cloned in pBluescript II SK and sequenced with its T7 and T3 primers.

Strains were inoculated on MS (mannitol 20 g, soya flour 20 g, agar 20 g, H_2_O to 1000 ml, pH7) medium covered with cellophane disks. After 2 days incubation at 42°C, the cells were fixed with fresh 2% glutaraldehyde (pH7.2) and 1% osmium tetroxide. Spores were examined with a JSM-6360LV scanning electron microscopy (Jeol).

### Isolation of plasmids from thermophilic *Streptomyces *strains

Isolating plasmid from thermophilic *Streptomyces *strains followed the protocol of Kieser [44] with sight modification. Strains were cultured in TSB liquid medium at 42°C overnight and mycelium was harvested by spinning at 4000 rpm for 15 min. About 50 μl mycelium was suspended in 350 μl TES buffer (25 mM Tris-HCL pH8, 25 mM EDTA pH8, 0.3 M sucrose, 2 mg/ml lysozyme, 5 μg/ml pre-boiled RNase A) and incubated at 37°C for 30 min. 44 μl of 10% SDS was added and mixed immediately by rotating and then 4 μl of 10 mg/ml proteinase K was added, followed by incubation for 60 min. 225 μl of 0.3 N NaOH/2% SDS was added and mixed immediately by vortexing, incubated at 70°C for 15 min and then cooled. 200 μl acid phenol/chloroform was added and vortexed and centrifuged at 12000 rpm for 10 min. The supernatant was transferred to a new centrifuge tube containing 55 μl un-buffered sodium acetate and 500 μl isopropanol was added. After mixing and centrifugation at 12000 rpm for 10 min and all liquid was removed using a pipette. The pellet was washed twice with 1 ml 70% ethanol, air dried and dissolved in 50 μl TE buffer.

### Growth curve of thermophilic *Streptomyces *strains in liquid culture

About 1.5 × 10^7 ^spores were inoculated into 50 ml TSB liquid medium supplemented with 0.01% antifoam289 (Sigma A 5551) and cultured at 30, 37, 45 and 50°C. 1 ml culture was harvested at each time-point and wet mycelium was harvested by centrifugation at 12000 rpm for 5 min. After drying for 10 min in a vacuum, the pellet was weighed with a fine balance (min. 10 mg). Growth curves were drawn with an average of three weighings at each time-point.

### Protoplast preparation and transformation of thermophilic *Streptomyces *strains

Protoplast preparation, regeneration and transformation of the thermophilic *Streptomyces *strains 2C and 4F followed standard *Streptomyces *protocols [6,45] with slight modifications. About 1 × 10^9 ^spores were inoculated into 50-ml YEME liquid medium (yeast extract powder 3 g, peptone 5 g, malt extract powder 3 g, glucose 10 g, with 25% sucrose, H_2_O to 1000 ml, pH7, supplemented with 0.5% glycine for 2C and 0.3% for 4F) at 45°C for ~7 h. Mycelium was harvested, washed once with 10.3% sucrose, and 1 mg/ml lysozyme solution in P buffer was added at 30°C (ca. 15 min for 2C and 30 min for 4F) to make protoplasts. After transformation, regeneration of protoplasts was achieved on R2YE medium at 45°C for ca. 9 h, to be selected by antibiotics.

### Construction of plasmids for transformation of thermophilic *Streptomyces *strains

Plasmids used in this work are listed in Table 2. Sizes of circular plasmids pTSC1, pTSC2 and pTSC3 and linear plasmid pTSL1 from thermophilic *Streptomyces *strains were measured by electrophoresis with known DNA markers (i.e. 1-kb supercoiled ladder and sequenced circular/linear plasmids). pQC156 [46] containing *Streptomyces *selection markers *melC/tsr *was cloned in an *E.coli *plasmid pSP72. *Kpn*I-treated pTSC1 was cloned in pQC156 to obtain pCWH1. The mesophilic *Streptomyces *replicons, including circular plasmids SCP2 [34], pFP1 and pFP11[33], linear plasmids SAP1 [36], pFRL2 [32] and pSHK1 [32], and integrating plasmid SLP1 [35], were cloned in pQC156 to yield pYQ40, pZR115, pZR10, pQC578, pHAQ61, pZR51, pGP9 and pZR205, respectively. These plasmids were introduced by protoplast transformation into thermophilic *Streptomyces *strains.

### Cloning and heterologous expression of the actinorhodin gene cluster in thermophilic *Streptomyces*

pHAQ31 [47] contained an *E.coli *replication origin and two *cos *sites of Supercos1 [48] and *Streptomyces *selection markers *melC/tsr *genes [31]. pHAQ31-derived cosmid N7-85 contained the whole actinorhodin biosynthetic gene cluster (5510413-5543521 bp) from *S. coelicolor *A3(2). A 3.4-kb *Xba*I/*Nhe*I fragment containing the phage фC31 integrase gene of pSET152 was cloned in a *Xba*I site of N7-85. The resulting plasmid, pCWH74, was introduced by conjugation from *E. coli *into thermophilic *Streptomyces *strains [38], which were cultured on R2YE (sucrose 103 g, K_2_SO_4 _0.25 g, MgCl_2_.6H_2_O 10.12 g, glucose 10 g, Difco Casaminoacids 0.1 g, trace element solution 2 ml, Difco yeast extract 5 g, TES 5.73 g, agar 22 g, H_2_O to 1000 ml, after autoclave and add 0.5% KH_2_PO_4 _5 ml, 5 M CaCl_2_.2H_2_O 4 ml, 20% L-proline 15 ml, 1N NaOH 7 ml) and MS media at 30, 37 and 45°C to detect blue actinorhodin pigment. To quantitate the production of actinorhodin, about 1 × 10^6 ^spores of M145 and 4F containing pCWH74 were inoculated into 50 ml R2YE liquid medium (lacking KH_2_PO_4 _and CaCl_2_) at 30 and 37°C; 1 ml culture was harvested in a time-course and treated with KOH, whereupon absorption at OD640 indicated actinorhodin production [39].

### Heterologous expression of the anthramycin biosynthetic gene cluster in thermophilic *Streptomyces*

An integrating cosmid, 024COA-3, containing the whole anthramycin biosynthetic gene cluster (EU195114.1, 1-33150 bp) (kindly provided by Prof. Brian Bachmann) was introduced by conjugation from *E. coli *into strain 4F [38]. Detection of anthramycin production followed Hu *et al. *[41]. After culturing in AP1 (corn starch 10 g, 2% peptonized milk, yeast extract powder 30 g, H_2_O to 1000 ml, pH7) medium at 47°C for 24 h, mycelium was extracted, dried and re-dissolved in MeOH. Anthramycin was first isolated on a HPLC column (Zorbax eclips 1.8 μm XDB-C18) and then mass spectrometry was performed using 6520 Agilent Accurate-Mass Q-TOF LC/MS. Anthramycin was separated by using a Zorbax eclips 1.8 μm XDB-C18 with a linear water-acetonitrile gradient containing 10 mM ammonium acetate (0.2 ml/min). The electrospray needle of the mass spectrometer was at 4000 V, the voltage of the skimmer was set to 65 V, Oct RF Vpp750V, collision ev 45 V, nebulizer pressure at 45 psig, and drying gas N2 350°C 9 L/min.

## Authors' contributions

WHC designed and performed all the experiments. ZJQ was involved in project design, and prepared the manuscript. All authors read and approved the final manuscript. The authors declare no conflict of interest.

## Supplementary Material

Additional file 1 **Predicted ORFs of plasmid pTSC1**. Detailed information and possible functions of the eight ORFs of pTSC1. Click here for file
