# Supplementary material for: Development of a gene cloning system in a fast-growing and moderately thermophilic Streptomyces species and heterologous expression of Streptomyces antibiotic biosynthetic gene clusters
Source: BMC Microbiol. 2011 Oct 28;11:243. doi: 10.1186/1471-2180-11-243 (PMC3212956; doi:10.1186/1471-2180-11-243)
Supplement: Additional file 1 — Predicted ORFs of plasmid pTSC1. Detailed information and possible functions of the eight ORFs of pTSC1. [file 1471-2180-11-243-S1.DOC]

**Table S1 Prediction of ORFs of thermophilic *Streptomyces* circular plasmid pTSC1**

| ORFs | Positions (bp) | Sizes (aa) | E. value | Possible functions |
| --- | --- | --- | --- | --- |
| pTSC1.1c | 220-41 | 59 | 2 × 10^-11^ | Hypothetical protein (*Streptomyces*) |
| pTSC1.2c | 1550-222 | 442 | 9 × 10^-76^ | Hypothetical protein (*Mycobacterium*) |
| pTSC1.3c | 2487-1984 | 167 |  | Unknown |
| pTSC1.4c | 2744-2511 | 77 | 6 × 10^-4^ | Hypothetical protein (*Streptomyces* plasmid pIJ101) |
| pTSC1.5c | 3594-3046 | 182 | 1 × 10^-55^ | SpdB (*Streptomyces* plasmid pIJ101) |
| pTSC1.6c | 5501-3591 | 636 | 0 | Tra (*Streptomyces* plasmid pIJ101) |
| pTSC1.7c | 5852-5607 | 81 | 8 × 10^-4^ | Hypothetical protein (*Streptomyces*) |
| pTSC1.8c | 6866-6513 | 117 | 5 × 10^-9^ | SpdA (*Streptomyces* plasmid pSNA1) |
